# Development and validation of the Social Emotional Assets (SEA) scale: a culturally responsive measure of social-emotional competencies in emerging adulthood

**DOI:** 10.3389/fpsyg.2026.1801087

**Published:** 2026-07-20

**Authors:** Kathleen Hughes

**Affiliations:** Department of Psychology, University of Calgary, Calgary, AB, Canada

**Keywords:** academic engagement, culture, emerging adulthood, personality, social emotional learning (SEL)

## Abstract

Social emotional competencies in children and youth are associated with academic and psychological wellbeing. However, application to post-secondary students has been limited, and measures have been centered on the white, middle-class experience. Emerging adulthood is a time of heightened emotionality, instability, and relationship vulnerability. Therefore, the development of social emotion competences in post-secondary students could promote healthy self-regulatory and interpersonal skills. The aim of this work was to develop a culturally sensitive measure of social and emotional competencies appropriate for use in emerging adulthood. A review of existing scales in social emotional learning and emotional intelligence was conducted. A total of 104 individual items from 4 scales were examined leading to the selection of the 35 items assessing SEA. A total of *N* = 2632 university students in western Canada were invited to participate in an anonymous online survey over a period of 4 years. This full data set was split into three independent samples for the current work. An exploratory factor analysis of the first subsample indicated a six-factor structure (empathy, cooperation, adaptability, awareness, assertiveness, prudence) of SEA. A confirmatory factor analysis of the second subsample supported this factor structure, and invariance analysis of group differences by ethnicity was supported at the configural, metric, and scalar level. Convergent and discriminant validity were examined using correlations and regressions in the third subsample. Factors of SEA were uniquely associated with individual HEXACO personality traits, and positively associated with perseverance, perceived teacher quality, academic engagement, and negatively associated with anxiety and Attention Deficit Hyperactivity Disorder (ADHD) symptoms. Hierarchical regression models indicated that components of SEA were found to be unique and significant predictors of academic engagement in university students. Further cross-cultural and longitudinal validation of this measure is required, particularly in diverse college and early adult populations. Results support the call for fostering SEA in higher education to benefit student mental health, healthy relationships and preparation for the labor market. Although many individuals develop SEA implicitly through social exposure, others may benefit from explicit instruction and intervention strategies.

## Introduction

Social and emotional competencies in children and youth are associated with academic and psychological wellbeing (Kim et al., 2024). The extent to which these skills continue to develop in emerging adulthood has been underexamined in the literature. Moreover, social developmental frameworks often lack cultural robustness and view social norms through a narrow lens of the majority (Li A. et al., 2025). Therefore, a goal of this work was to develop a culturally sensitive measure of social and emotional competencies appropriate for use in emerging adulthood.

Although research on character development in developmental psychology has been ongoing since the work of Kohlberg (Kohlberg, 1984), social and emotional competencies gained focus after Gardner's (2011) inclusion of interpersonal and intrapersonal intelligence in his theory of multiple intelligences which then influenced Goleman's (1995) work on emotional intelligence. This theoretical work was adapted for educators by the Collaborative for Academic, Social, and Emotional Learning (CASEL; Elias et al., 1997).

CASEL defined their work as focused on social and emotional learning (SEL) which was defined as the process in which individuals develop the skills, values, and attitudes necessary for social and emotional competence (Elias et al., 1997). SEL could be promoted in schools by fostering supportive and safe learning environments, promoting moral and ethical thinking, mentoring problem solving and critical thinking skills, encouraging inclusion and cross-cultural perspectives, and connecting learning to larger community needs. Outcomes of SEL were conceptualized to include competency in expressing and managing emotions, perspective taking, cooperation, goal setting, relationship skills, self-regulation, and increased school attendance (Elias and Weissberg, 2000).

SEL has significant short-term and long-term effects (Durlak et al., 2011; Merrell et al., 2008). Immediately after an intervention, SEL was associated with decreased behavioral problems and increased academic performance, student engagement, and attachment to school. Years beyond an intervention, SEL was connected with higher graduation rates, increased career success, lower substance abuse, lower criminal activity, and prevention of harm to the self and others (Denham, 2018).

In early childhood, SEL is associated with school readiness and executive function (Stefan et al., 2022). At middle childhood, SEL is connected to understanding socially acceptable behaviors and peer acceptance (Bierman and Motamedi, 2015). By adolescence, SEL is linked with positive mental health development, effective communication, and the development of healthy relationships (Kim et al., 2024).

Notably, SEL is predictive of longitudinal life-long success in job markets, relationships, mental health, and cognitive abilities (Shi and Cheung, 2024), leading to the advocacy of SEL instruction in K-12 educational programs (Bierman and Motamedi, 2015). However, the continuation of SEL development into adulthood has been underexamined (Conley, 2015), with majority of works largely theoretical or qualitative in nature (Elmi, 2020; Yeh et al., 2022). One recent study has found an SEL intervention amongst 40 university students was positively associated with academic engagement and negatively associated with academic stress (Vargas-Madriz and Konishi, 2025).

Emerging adulthood is a time of heightened emotionality, instability, and relationship vulnerability (Arnett, 2024). As emerging adulthoods gain independence, many struggle with new responsibilities, roles, and life decisions (Katsiaficas, 2017). In addition, stressors, such as financial and employment insecurity, relationship instability, and academic performance peak in emerging adulthood (Ng et al., 2024). Over 62% of clinical psychological disorders are diagnosed by age 25 (Solmi et al., 2022). Therefore, the development of social emotion competences in post-secondary students could promote healthy self-regulatory and interpersonal skills (Tabassum et al., 2020).

Growth in research on SEL has led to complexities in how the construct is conceptualized. In a review of over 60 intervention programs, subcomponents, predictors, and outcomes of SEL were found to overlap (Stefan et al., 2022). For instance, prosocial behavior is identified as a predictor, a component, and an outcome of SEL depending on the study (Jackson et al., 2024). In addition, there is incongruence in the literature whether SEL is the process in which skills are developed, or the skills and competencies themselves (Kim et al., 2024).

Assessment paradigms of social and emotional competencies are often not standardized. Assessments include the use of single skill measures (i.e., perseverance, regulation, or perspective taking in isolation; Jackson et al., 2024), or inappropriate tools such as personality scales (McAbee et al., 2019; OECD, 2018) and business management inventories (Poonamallee et al., 2018). Lexical approaches to trait theories of personality often start broad, examining all possible characteristics that can define differences in individuals (Costa and McCrae, 1995), and focus on many stable, enduring traits that are considered dispositional rather than competency based (Ashton and Lee, 2007).

SEL assessments, intervention frameworks, and theoretical definitions have been built using a white, middle-class, American worldview (McCall et al., 2023). Although the original definition of SEL specified the inclusion of cross-cultural perspectives, this element has been limited to the translation of texts, and diverse imagery in materials. SEL frameworks have been used cross culturally in Portugal (Coelho et al., 2023), Ghana (Ntum et al., 2025), China (Chen et al., 2025) and Hispanic communities (Yang and Golshirazi, 2024), and cultural and ethnic differences have emerged. In particular, scores on the LeBuffe et al. (2014) demonstrate an advantage for children of European heritage, and a disadvantage for children of African and Hispanic heritage (Lawson, 2023). One possible explanation for this disparity is that assessment focuses on culturally specific and class specific skills and therefore may have an inherent cultural and class bias. Social norms and encouraged behaviors that are adaptable to cultural values are lacking in SEL work (Li A. et al., 2025).

Despite cultural and ethnic differences in assessment, SEL interventions have been found to be especially beneficial for immigrant youth as compared to non-immigrant adolescents (Yang and Golshirazi). Moreover, SEL is associated with increased academic engagement and reduced stress and burnout in both Ghanian and Chinese children (Chen et al., 2025; Ntum et al., 2025). Further development of SEL tools that are culturally responsive may assist individuals with navigating larger societal and social processes, build resilience, and foster connection across race, class, and culture (McCall et al., 2023).

A goal of the current work was to address the limitations in conceptualization, assessment, developmental appropriateness, and cultural robustness in social and emotional competencies. For the purpose of this work, SEL is considered to be the process of developing social and emotional competencies via educational materials, and intervention strategies. Assessment and measurement of social and emotional competencies is considered distinct from SEL and will be referred to as Social Emotional Assets (SEA). These are both separate from personality, which is considered to be enduring patterns of thoughts, feelings, and behaviors across the lifespan (Costa and McCrae, 1995).

SEA is defined as a composite of multiple skills relating to self-regulation and interpersonal interactions. These skills are malleable, developing over the lifespan, and distinct from personal disposition or personality. Whereas many individuals learn these competencies implicitly and through their culture, others may benefit from direct, explicit instruction and intervention (Merrell et al., 2008).

Although currently existing measures of SEA tend to focus on childhood and adolescence, much of our social and emotional development occurs in the adult years. Social competence, self-regulation, and interpersonal wisdom continue to develop throughout the lifespan (Rakoczy et al., 2018). Therefore, there is a need for a measure of SEA that extends beyond specific K-12 grades and includes items that are appropriate for emerging adulthood. To examine the convergent and discriminant validity of this new measure, indices of personality and disposition were also examined. To examine the predictive validity of the SEA measure, indices of psychological and academic wellbeing were also collected. In particular, self-reports of symptoms of anxiety and ADHD along with student engagement were examined. ADHD and anxiety are two prevalent and divergent forms of student mental distress in university which have been associated with decreased student engagement (Barth et al., 2025).

The goal of this study was to build and validate a culturally robust measure of SEA that was theoretically derived and empirically substantiated. This scale development included following best practices outlined in psychometric literature such as item generation, theoretical analysis, extraction of factors, and tests of dimensionality, reliability, and validity (Morgado et al., 2017; Boateng et al., 2018).

It terms of scale development, it was hypothesized that items would load on a five-factor structure similar to the CASEL model (self-awareness, self-regulation, social awareness, relationship skills, responsible decision-making). In addition, it was predicted that the factor-structure would be confirmed for both white and ethnic minority students.

It was hypothesized that subcomponents of SEA would be strongly inter-associated with other SEA factors but uniquely associated with personality characteristics such as HEXACO traits and perseverance. It was also hypothesized that higher SEA would be associated with increased psychological and academic wellbeing. Finally, it was predicted that after controlling for perseverance and psychological wellbeing, SEA would be a unique predictor of academic engagement in emerging adulthood, which would replicate findings found in K-12 students (Denham, 2018).

## Methods

### Participants

Over a 4 year period, a total of *N* = 2632 undergraduate students enrolled in introductory psychology in western Canada participated in this study. These students ranged in age from 17 to 26 years old (*M* = 22.4, *SD* = 2.65). The majority of this sample were women (82%) with some men (16%) and non-binary (2%) participants. Sexual minorities made up 16% of the sample. Approximately half of the sample were white (54%), with multiple minority groups represented such as South Asian (14%), East Asian (9%), South East Asian (8%), Middle Eastern (5%), Black (3%), Hispanic (3%), and Indigenous (2%).

### Procedure

Data were collected as part of four independent undergraduate thesis projects, which were approved by the university's Conjoint Faculties Research Ethics Board (REB17-1953; REB18-0940; REB19-0940; REB22-1490). In each study, introductory psychology students were invited to complete an anonymous online survey hosted on Qualtrics for course credit (1% of final grade). Students were given informed consent and told that participation was voluntary, and they would withdraw their participation at any time and still receive course credit. All participants were asked to confirm consent a second time at the end of the survey and then received debriefing with contact numbers for wellness centers on campus. Participants in one study were excluded from participation in all subsequent studies to prevent duplication of data.

### Measures

#### SEA measurement development

A review of existing scales in SEL, social skills, and emotional intelligence was conducted. A total of 104 individual items from four scales were examined, which included the Devereux Student Strengths Assessment (DESSA; LeBuffe et al., 2014); Emotional and Social Competency Inventory (ESCI; Boyatzis and Goleman, 2007); Panorama (Panorama Education, 2020); and Social Skills Improvement System—Social Emotional Learning Edition (SSIS-SEL; Gresham and Elliott, 2017). Redundant items were removed.

Of the remaining 68 items, a focus group of 12 university students from different cultural backgrounds discussed each item for its (1) relevance to self-regulatory or interpersonal competencies, (2) cultural universality, and (3) age appropriateness in emerging adulthood. Six items were considered to be more focused on metacognition and work performance rather than SEA (e.g., “I know which skills I perform well and which skills I do not”). Eighteen items were discussed and determined to be culturally exclusive due to differences in social norms and values (e.g., “I look at people when I talk to them”). Finally, nine items were considered to be developmentally inappropriate for emerging adulthood (e.g., “I follow directions in class”). The full list of removed items are displayed in Appendix A.

This left a total of 35 items. The Social Emotional Assets (SEA) scale was created as a self-report measure consisting of these 35 items. Participants were instructed to rate how much they agreed with each statement on a five point Likert scale with 1 = strongly disagree, 2 = disagree, 3 = neutral/unsure, 4 = agree, and 5 = strongly agree.

#### Personality and disposition

##### Personality

Due to personality being used as a proxy measure for social emotional competencies (e.g., McAbee et al., 2019), the association between personality and SEA was investigated. Personality was assessed with the HEXACO 60-item Inventory (Lee and Ashton, 2004) which is considered to be a culturally inclusive measure of personality (Tosi et al., 2025). The HEXACO is a 5-point Likert scale that contains six subscales of personality (Humility-Honesty, Emotionality, Extraversion, Agreeableness, Conscientiousness, and Openness) which are considered to be enduring and dispositional over the lifespan. The HEXACO is one of the leading instruments of personality and has been cited over 3,000 times and used for over 20 years (Ashton and Lee, 2020).

##### Perseverance

Unlike SEA, traits such as effortful control, grit, and perseverance are considered to be temperamental and are reliably stable over the lifespan (Sigmundsson, 2021). Perseverance was measured using the Grit Scale (Duckworth et al., 2007) which is a 12 item self-report scale of one's continued perseverance of long-term goals. This measure has been validated with cross-cultural samples (Jaramillo-Rincon et al., 2024).

#### Psychological wellbeing

##### Anxiety

To examine the association between SEA and psychological wellbeing, anxiety was assessed with the General Anxiety Disorder seven-Scale (Mills et al., 2014). This self-report measure assesses anxiety symptoms on a five-point Likert scale and has been found to have reasonable reliability and validity (Dhira et al., 2021).

##### ADHD

An alternate measure of psychological wellbeing in this work was ADHD symptomology via the Adult ADHD Self-Report Scale (Kessler et al., 2005). ADHD in university students can impact student engagement (Barth et al., 2025). This is an 18-item self-report measure of inattention, carelessness, and physical restlessness. This measure has been validated for post-secondary students (Lee and Zhang, 2025).

#### Academic wellbeing

##### Teacher quality

To examine the association between SEA and academic wellbeing, educational satisfaction was assessed via a measure of student-rated teacher quality. This was measured using the Motivational Instructional Contexts Inventory (Law, 2011) which is a student-rated, 14 item scale of professor's supportiveness and clarity of instruction. Although this measure was originally used for K-12 students (Li L. et al., 2025), items were adjusted to reflect a post-secondary learning environment (i.e., changing teacher to professor).

##### Student engagement

Student engagement was assessed using a multifaceted, 27 item measure that was validated in 12 countries (Lam et al., 2014). This measure assesses three subcomponents of engagement including behavioral engagement “*I pay attention in class*,” emotional engagement “*I am very interested in learning,”* and cognitive engagement “*I make up my own examples to understand better*.” Student engagement is a predictor of academic achievement (Hughes and Coplan, 2018). Items on this measure were adjusted to better reflect a post-secondary learning environment (i.e., changing classroom environment to campus environment, school to university). Overall, this measure has been found to be a reliable predictor of academic adjustment (Zhang and Liu, 2025).

## Results

### Data splitting

The complete data of *N* = 2632 was split into three separate subsamples using SPSS version 29. A random number generator was used to create a case number variable, the dataset was then sorted by this random number and then sorted into thirds. Due to missing data and participant attrition, subsamples were not identical in size, but similar. Crosstabs were conducted to examine differences between the subsamples in terms of gender, ethnicity, and sexuality, and Multivariate Analysis of Variance (MANOVA) was conducted to evaluate possible differences in any continuous study variables. All results were non-significant [*WilksLambdaF*_(44, 1, 610)_ = 0.87, *p* = 0.72, *partial* η^2^ = 0.02, *power* = 0.92]. A data plan was followed in which subsample 1 (*N* = 873) was used to conduct an exploratory factor analysis of the SEA measure; subsample 2 (*N* = 878) was utilized for a confirmatory factor analysis of the SEA measure; subsample 3 (*N* = 881) was for convergent, and discriminant validity of the SEA measure.

### Exploratory factor analysis

An exploratory factor analysis (EFA) was conducted to examine the inter-associations between the 35 items on the SEA scale for subsample 1 (*N* = 873). As the factors of SEA were theoretically conceptualized to be orthogonal, a varimax rotation was used (Worthington and Whittaker, 2006). Eight factors emerged with eigenvalues greater than 1, which was also supported by a scree plot analysis. These eight factors accounted for 52% of the variance across the items. Factor loadings with a threshold of 0.33 indicated that seven items were found to cross load, and three factors were considered problematic. Factors 6 and 7 had only two items focusing on future planning and gratification delay, respectively. As both of these concepts were similar to the CASEL factor of responsible decision making, they were combined tentatively (Elias et al., 1997). Factor 8 contained three items, which were all cross-loaded, two of which with Factor 2. Items on Factors 2 and 8 were similar to the CASEL factor of self-regulation. Therefore, Factor 2 and 8 were combined tentatively. A all factors and their items, along with cross-loaded items are displayed in Table 1.

**Table 1:** EFA rotated component matrix for Social Emotional Assets (SEA) scale.

Item	1	2	3	4	5	6	7	8
I try to help when others are sad	0.75							
I care deeply about other people's feelings	0.74							
I try to think about how others feel	0.72							
I compliment other's accomplishments	0.61							
I resolve conflicts by addressing people's perspectives and feelings	0.53							
I listen carefully to other people's points of view	0.52							
I try to forgive others when they say sorry	0.42							
I know I can get through any challenge		0.64						
When I'm stressed, I'm able to calm down		0.64						
I can control my emotions when I need to		0.62	0.32					
I am an optimistic and hopeful person		0.55						
I can remain calm, even when someone is bothering me		0.50	0.48					
I can disagree with others without starting an argument			0.66					
I stay calm when people point out my mistakes			0.53					
I cooperate with those who are different than me			0.52					
I do my work without bothering others			0.48			0.35		
When I am competing against someone, I am polite and respectful	0.38		0.42					
I can stand up for myself without putting others down			0.36					
I am aware of my own feelings				0.75				
I know right away when something is bothering me				0.66				
I am able to recognize when I am stressed				0.62				
I can describe underlying reasons for my own feelings		0.34		0.61				
I understand how my emotions can impact my behaviors	0.36			0.45				
When I need help, I am comfortable asking for assistance					0.83			
I ask for help when I need it					0.82			
I ask for feedback even when I know it may not be positive					0.52			
I say nice things about myself without bragging					0.38			
I think about my future when I make decisions						0.76		
I consider the outcomes before I act						0.71		
I can wait for good things to happen							0.72	
Waiting to earn a reward or have a treat is easy for me							0.66	
I adapt to shifting priorities and rapid change		0.48						0.54
I am comfortable giving an opinion when asked					0.34			0.53
I adjust well to changes in plans		0.47						0.48

*N* = 873.

Factor 1, had seven items, “*I care deeply about other people's feelings*,” “*I try to help when others are sad*,” “*I try to think about how others feel*,” “*I help my friends when others are in need*,” “*I compliment other people's accomplishments*,” “*I resolve conflicts by addressing people's perspectives and feelings*,” and “*I try to forgive others when they say sorry.”* This factor was one of three factors similar to CASEL's concept of relationship skills.

After combining Factors 2 and 8, the next factor had five items, “*When I'm stressed, I'm able to calm down*,” “*I am an optimistic and hopeful person*,” “*I know I can get through any challenge*,” “*I adjust well to changes in plans*,” and “*I adapt to shifting priorities and rapid change.”* This factor was most similar to CASEL's concept of self-regulation.

Factor 3 had four items, “*I stay calm when others point out my mistakes,”* “*I can disagree with others without starting an argument*,” “*I cooperate with those who are different from me*,” and “*I can stand up for myself without putting others down.”* This was the second factor which overlapped with CASEL's concept of relationships skills.

Factor 4, had three items, “*I am aware of my own feelings*,” “*I know right away when something is bothering me*,” “*I am able to recognize when I am stressed.”* This factor was most similar to CASEL's concept of self-awareness.

Factor 5, had four items, “*I ask for feedback even when I know it may not be positive,”* “*When I need help, I am comfortable asking for assistance,”* “*I ask for help when I need it*,” and “*I say nice things about myself without bragging*.” This was the third factor which overlapped with CASEL's concept of relationship skills.

After combining Factors 6 and 7, the final factor had four items, “*I think about the future when I make decisions,” “I consider the outcomes before I act,” “I can wait for good things to happen,” “Waiting to earn a reward or have a treat is easy for me.”* This factor was most similar to CASEL's concept of responsible decision making.

Of the 7 cross-loaded items, two loaded on both Factor 2 and 3, “*I can control my emotions when I need to*,” and “*I can remain calm, even when someone is bothering me*.” Among the other five items, one item cross-loaded on Factor 3 and 6 *(“I do my work without bothering others*,”) one on Factor 1 and 3 (“*When I am competing against someone, I am polite and respectful*,”), one on Factor 2 and 4 (“*I can describe underlying reasons for my own feelings*,”), one on Factor 1 and 4 (“*I understand how my emotions can impact my behaviors*,”), and one on Factor 4 and 5 (“*I am comfortable giving an opinion when asked*”).

### Confirmatory factor analysis

**Figure 1:**
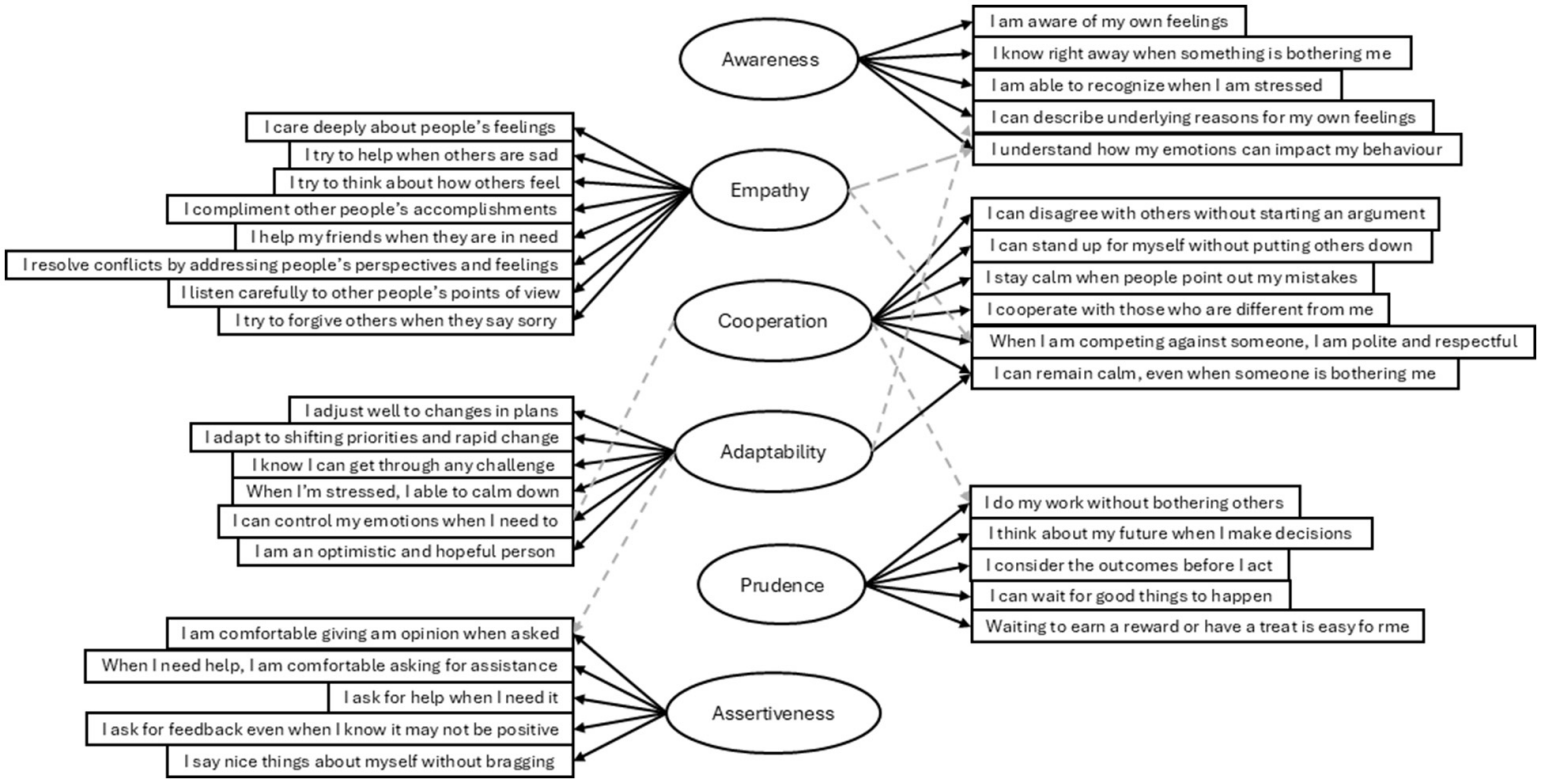
Confirmatory factor analysis structure of the Social Emotional Assets (SEA) scale. *N* = 878.

A confirmatory factor analysis (CFA) was conducted with Amos software 27 (AMOS, IBM, New York, NY) and subsample 2 (*N* = 878). Six latent variables were drawn according to the factor loadings in the preceding EFA. Cross-loaded items were drawn as connecting to two latent variables. Error terms for items on the same factor were drawn to covary, and one regression weight on each factor was set to 1 (Kline, 2023). Latent factors were drawn to covary with each other. The final model is shown in Figure 1.

The overall model was found to have acceptable fit [$X_{\left(467\right)}^{2}$ = 1178, *p* < 0.001, Tucker-Lewis Index (TLI) = 0.87, Comparative Fit Index (CFI) = 0.92, Root Mean Square Error of Approximation (RMSEA) = 0.035]. Notably, six of seven cross-loaded items in the EFA were found to only load on 1 factor in the CFA. Significant and non-significant estimates are shown in Figure 1. The remaining cross-loaded item “*I can remain calm, even when someone is bothering me*,” was dropped from the measure. Overall, this analysis indicated a six-factor solution which divergent significantly from the CASEL framework. In particular, three factors concerning relationship skills were named Empathy (eight items), Cooperation (five items), and Assertiveness (five items). One factor relating to self-regulation was named Adaptability (six items), one factor relating to self-awareness was named Awareness (five items), and the final item relating to responsible decision making was named Prudence (five items).

Reliability of factors was examined using JASP software (JASP, University of Amsterdam, Amsterdam) to calculate Cronbach's alpha and McDonald's omega in subsample 2 (Dunn et al., 2014). Although alpha and omega were similar for each factor, reliability for individual factors ranged from strong to modest to weak. (Empathy α = 0.81, ω = 0.81; Adaptability α = 0.76, ω = 0.76; Assertiveness α = 0.69, ω = 0.70; Awareness α = 0.69, ω = 0.69; Cooperation α = 0.62, ω = 0.62; Prudence α = 0.56, ω = 0.57).

### Ethnicity and gender effects

To examine cultural bias of the SEA scale invariance analysis was conducted using the multiple groups tool in AMOS software version 27 using split sample 2. The analysis compared the factor structure of SEA amongst white majority students (*n* = 394) to ethnic minority students (*n* = 402). Although the configural model showed weaker fit than the full-sample model, changes in fit indices supported metric and scalar invariance (Putnick and Bornstein, 2016). Residual invariance was not retained because ΔCFI exceeded the recommended threshold of.010. Invariance analysis details are shown in Table 2.

**Table 2:** Invariance analysis of ethnicity.

Model	TLI	CFI	ΔCFI	RMSEA	ΔRMSEA
Configural	0.830	0.879	—	0.034	—
Metric	0.834	0.873	0.006	0.034	0.000
Scalar	0.834	0.869	0.004	0.034	0.000
Residual	0.836	0.855	0.014	0.034	0.000

Configural = equivalent factor structure, Metric = constrained factor loadings, Scalar = constrained item intercepts, Residual = constrained item residuals.

*N* = 878.

Gender invariance could not be tested as split sample 2 only included *n* = 147 men, which was insufficient sample size for stable multi-group CFA estimation. To examine gender differences in SEA, a MANOVA was conducted comparing women (*n* = 692) and men (*n* = 147). This model was found to be significant [Wilks' Lambda *F*_(6, 832)_ = 5.94, *p* < 0.001, partial η^2^ = 0.04, power = 1.00]. Univariate results indicated significant gender differences in adaptability [*F*_(1, 837)_ = 18.67, *p* < 0.001, partial η^2^ = 0.02, power = 0.99], with men (*M* = 3.75, *SD* = 0.65) reporting higher scores than women (*M* = 3.49, *SD* = 0.67); prudence [*F*_(1, 837)_ = 9.54, *p* < 0.01, partial η^2^ = 0.01, power = 0.87], with men (*M* = 4.01, *SD* = 0.45) reporting higher scores than women (*M* = 3.87, *SD* = 0.53); and assertiveness [*F*_(1, 837)_ = 4.81, *p* < 0.05, partial η^2^ = 0.01, power = 0.59], with men (*M* = 3.67, *SD* = 0.64) reporting higher scores than women (*M* = 3.52, *SD* = 0.65). Finally, there was a significant gender effect found in empathy [*F*_(1, 837)_ = 3.92, *p* < 0.05, partial η^2^ = 0.01, power = 0.51], with women (*M* = 4.23, *SD* = 0.44) reporting higher scores than men (*M* = 4.18, *SD* = 0.44). No significant gender effects were found for cooperation or awareness.

### Descriptive statistics

Planned missing data resulted in disparities in sample sizes across study variables, with fewer participants receiving survey questions about ADHD symptoms, cognitive engagement, and personality as compared to perseverance, anxiety, and behavioral engagement. This was due to the nature of data collection, which was collected over several years as a part of 4 independent undergraduate thesis projects, each including different variables. No data imputation or replacement procedures were employed. Notably, a MANOVA examining cohort effects was significant [Wilks' Lambda *F*_(18, 7, 306)_ = 6.35, *p* < 0.001, partial η^2^ = 0.02, power = 1.00]. Tukey's Least Significant Difference *post hoc* indicated the first chronologically collected sample self-reported lower levels of SEA as compared to all subsequent samples (e.g., Assertiveness *M* = 3.39 as compared to 3.60 for other groups).

Missing data at the item-level within a survey instrument were less than 2% was uncorrelated with study variables and demographics and therefore considered to be non-systematic and potentially Missing at Random (Schafer and Graham, 2002). Summary scores for study variables were calculated by taking the weighted mean of item scores for participants who completed over 50% of the scale or subscale items. This resulted in less than 1% missing data at the summary score level within a given cohort.

Descriptive statistics for all study variables for subsample 3 are shown in Table 3. Overall, study variables were found to follow a normal distribution, however some measures were leptokurtic such as student-perceived teacher quality. Means and standard deviations show that the typical study participant considered themselves to be empathetic and self-aware, high in SEA and conscientiousness, and moderately low in ADHD symptomology.

**Table 3:** Study variable descriptive statistics.

Variable	*N*	M	SD	Min-Max	Skewness	Kurtosis	α/ω
SEA							
Empathy	863	4.26	0.46	2.3–5.0	−0.46	0.21	0.82/0.82
Cooperation	869	3.88	0.48	1.7–5.0	−0.39	0.79	0.63/0.63
Adaptability	863	3.52	0.60	1.2–5.0	−0.42	0.08	0.74/0.74
Awareness	870	4.03	0.55	1.4–5.0	−0.58	0.86	0.71/0.71
Assertiveness	869	3.73	0.61	1.0–5.0	−0.37	0.09	0.68/0.70
Prudence	868	3.89	0.49	1.80–5.0	−0.46	0.85	0.55/0.55
Personality							
Honesty	429	3.35	0.65	1.3–5.0	−0.15	−0.19	0.76/0.77
Emotionality	429	3.57	0.62	1.2–5.0	−0.35	0.09	0.73/0.74
Extraversion	434	3.11	0.68	1.1–5.0	−0.12	−0.18	0.77/0.79
Agreeableness	434	3.33	0.60	1.3–5.0	−0.23	0.17	0.76/0.77
Conscientiousness	433	3.60	0.57	1.8–5.0	−0.28	−0.30	0.74/0.74
Openness	434	3.36	0.66	1.4–5.0	−0.16	−0.33	0.77/0.78
Anxiety	706	3.30	0.77	1.0–5.0	−0.19	−0.42	0.73/0.73
ADHD	287	2.85	0.48	1.3–4.8	0.38	0.41	0.82/0.82
Perseverance	706	3.39	0.63	1.3–5.0	0.42	0.12	0.81/0.84
Teacher quality	706	3.46	0.53	1.2–5.0	−0.58	1.06	0.89/0.89
Engagement							
Behavioral	704	3.41	0.53	1.6–5.0	−0.02	0.06	0.72/0.68
Cognitive	706	3.79	0.53	1.3–5.0	−0.38	0.68	0.80/0.80
Emotional	706	3.94	0.52	1.3–5.0	−0.47	0.77	0.81/0.81

*N* = 881.

### Correlations

To assess the convergent and discriminant validity of the SEA scale, correlations between each SEA factor with personality, perseverance, psychological and academic wellbeing were examined using subsample 3 (see Table 4). Overall, study variables were highly inter-associated. Of note, SEA factors were uniquely associated with HEXACO personality traits. For instance, empathy and cooperation had the largest correlation coefficient with agreeableness, whereas adaptability and assertiveness were most highly correlated with extraversion, and awareness and prudence were most highly correlated with conscientiousness. Overall, the factors of SEA were positively associated with perseverance, student-perceived teacher quality, and academic engagement, and negatively associated with anxiety and ADHD symptoms.

**Table 4:** Correlations of SEA factors to all study variables.

Variable	Empathy	Cooperation	Adaptability	Awareness	Assertiveness	Prudence
Honesty	0.35^***^	0.27^***^	0.11^*^	0.10^*^	0.01	0.21^***^
Emotionality	0.27^***^	−0.12^*^	−0.31^***^	0.16^***^	0.03	−0.03
Extraversion	0.21^***^	0.20^***^	0.49^***^	0.21^***^	0.50^***^	0.16^**^
Agreeableness	0.32^***^	0.46^***^	0.37^***^	0.08	0.14^**^	0.24^***^
Conscientiousness	0.22^***^	0.16^**^	0.14^**^	0.30^***^	0.27^***^	0.37^***^
Openness	0.19^***^	0.19^***^	0.05	0.05	0.05	0.13^**^
Anxiety	−0.01	−0.16^***^	−0.52^***^	−0.07	−0.24^***^	−0.14^***^
ADHD	−0.06	−0.11	−0.26^***^	−0.19^**^	−0.21^***^	−0.35^***^
Perseverance	0.11^**^	0.14^***^	0.24^***^	0.20^***^	0.17^***^	0.24^***^
Teacher quality	0.22^***^	0.23^***^	0.29^***^	0.25^***^	0.27^***^	0.23^***^
Behavioral Eng.	0.29^***^	0.27^***^	0.26^***^	0.23^***^	0.31^***^	0.29^***^
Emotional Eng.	0.36^***^	0.28^***^	0.35^***^	0.30^***^	0.30^***^	0.29^***^
Cognitive Eng.	0.39^***^	0.36^***^	0.37^***^	0.30^***^	0.34^***^	0.34^***^

^*^p < 0.05, ^**^p < 0.01, ^***^p < 0.001.

*N* = 881.

### Hierarchical regressions

To investigate the utility of SEA as a unique predictor of student engagement, a series of three hierarchical regression models were conducted. In each model, teacher quality, anxiety, and perseverance were entered in Step 1. Originally, ADHD was planned to be entered in Step 1, however due to planned missing data, ADHD scores were only available for 32% of the subsample 3 participants and therefore was excluded from the analysis. All six subscales of SEA were entered in Step 2. Multicollinearity diagnostics were acceptable across all models (Tolerance = 0.51–0.88; Variance Inflation Factor (VIF) = 1.38–1.97). Standardized betas, *t* statistic, and significant levels for each predictor across three regression models are presented in Table 5. Model fit statistics are reported in text below.

**Table 5:** Hierarchical regression results: standardized betas of predictors and outcomes.

Variable	Behavioral engagement		Emotional engagement		Cognitive engagement	
	*t*	β	*t*	β	*t*	β
Teacher quality	3.81	0.12^***^	10.78	0.35^***^	2.59	0.10^*^
Anxiety	1.67	0.06	1.17	0.04	4.77	0.21^***^
Perseverance	17.42	0.53^***^	7.28	0.23^***^	2.71	0.10^**^
Assertiveness	4.55	0.15^***^	1.49	0.05	3.20	0.13^**^
Awareness	−1.03	−0.04	0.91	0.03	0.40	0.02
Adaptability	−0.14	−0.01	3.13	0.13^**^	5.09	0.25^***^
Empathy	3.80	0.13^***^	5.11	0.19^***^	4.43	0.19^***^
Cooperation	1.14	0.04	−0.46	−0.02	1.07	0.05
Prudence	1.50	0.05	0.83	0.03	2.08	0.09^*^

^*^p < 0.05, ^**^p < 0.01, ^***^p < 0.001.

*N* = 881.

The first model, predicting behavioral engagement, was significant [*F*_(9, 688)_ = 59.61, *p* < 0.001] and accounted for a strong amount of variance [*Adjusted R*^2^ = 0.43, Δ*R*^2^ = 0.06, Δ*F*_(6, 688)_ = 12.32, *p* < 0.001]. Assertiveness and empathy were found to be unique predictors of behavioral engagement. The second model, predicting emotional engagement was significant [*F*_(9, 688)_ = 8.22, *p* < 0.001] and accounted for a moderate amount of variance [*Adjusted R*^2^ = 0.38, Δ*R*^2^ = 0.07, Δ*F*_(6, 688)_ = 13.83, *p* < 0.001]. Adaptability and empathy were found to be unique predictors of emotional engagement. The third model, predicting cognitive engagement was significant [*F*_(9, 547)_ = 27.68 *p* < 0.001] and accounted for a moderate amount of variance [*Adjusted R*^2^ = 0.30, Δ*R*^2^ = 0.21, Δ*F*_(6, 547)_ = 27.51, *p* < 0.001]. In this model, assertiveness, adaptability, empathy, and prudence were found to be significant predictors of cognitive engagement.

## Discussion

The goal of this work was to design and validate a culturally sensitive measure of Social Emotional Assets (SEA) in emerging adulthood. As many available measures of SEA are directed at children and adolescents (LeBuffe et al., 2014) and from limited cultural perspectives (McCall et al., 2023), this new measure sought to address both those concerns.

A 34 item scale was developed which contained a six-factor structure (empathy, cooperation, adaptability, awareness, assertiveness, prudence). Notably, this structure did not replicate the theoretical five factor structure of CASEL (self-awareness, self-regulation, social awareness, healthy relationships, responsible decision-making; Durlak et al., 2011). In a review of the original 104 items considered for the measure, items related to social awareness were largely omitted due to the criteria of cultural universality. For instance, “*I look at people when I talk to them,”* and “*I understand the reasons for others' actions*,” both refer to understanding and navigating social interactions, but are potentially biased against ethnic minorities given that eye contact in conversation (McCarthy et al., 2006) and the attribution style of understanding others' motivations are both culturally relative constructs (Krull et al., 1999). Moreover, items related to healthy relationships were expanded and loaded onto three factors of empathy (e.g., “*I try to think about how others feel*,”), cooperation (e.g., “*I can disagree with others without starting an argument*”), and assertiveness *(“When I need help, I am comfortable asking for assistance*”). This supports previous work that found developing mature and supportive relationships is a key milestone in emerging adulthood (Chopik et al., 2022).

Invariance analysis supported the factor structure for White and ethnic minority students to the scalar level. This addresses a focal concern over the cultural bias and prioritization of white, middle-class experiences in previous measures (Lawson, 2023; Li A. et al., 2025). As scale items were reviewed for cultural universality by a focus group, this may have reduced cultural bias. Additional recommendations made by the focus group were to modify but not delete some items to support cultural universality. For instance, the item, “*I look at people when I talk to them*,” was dropped from the final measure, an alternative approach would be to replace the item with, “*I make appropriate eye contact for a given situation and context.”*

Women rated themselves higher in empathy as compared to men, and men rated themselves higher in assertiveness and adaptability. This reflects meta-analyses that men tend to choose more assertive language than women (Leaper and Ayres, 2007; Manian and Sheth, 2021). Moreover, this supports neurobiology theories that women display higher levels of empathy due to ethological as well as cultural reasons (Christov-Moore et al., 2014).

Components of social emotional learning (SEL) have been conflated with predictors of SEL and with dispositional traits (Jackson et al., 2024; McAbee et al., 2019). This work sought to articulate the differences between malleable social and emotional competences, and stable personality traits. Although personality can shift and mature over the lifespan, it is considered to be less skills-based and more temperamental (Lee and Ashton, 2004). Moreover, many components of personality are preference based and not competency based (Costa and McCrae, 1995), and identifying a personality constellation as adaptive or maladaptive ignores the importance of goodness-of-fit and environmental niche (Lahdelma et al., 2021).

Differential correlations between subcomponents of SEA and HEXACO support the divergent validity of the six SEA factors. In the current study, high emotionality was negatively associated with cooperation and adaptability but positively associated with empathy, which indicates that high emotionality could be considered an asset and a drawback in different circumstances (Lee, 2009). High honesty-humility was associated with empathy and cooperation, but not assertiveness or adaptability. This may suggest that although cooperative, individuals high in honesty-humility have difficulty advocating for themselves (Li L. et al., 2025). In comparison, agreeableness was associated with empathy, cooperation and adaptability, which may reflect that being agreeable is amenable to changes in one's circumstances (Yang et al., 2020). Finally, extraversion was strongly correlated with adaptability and assertiveness, which reflects the subfactors of extraversion such as boldness and liveliness (Malcolm et al., 2021).

Convergent validity can be inferred from the similar associations of SEA and other study variables. Notably, all six factors of SEA were positively associated at similar strengths to perseverance, perceived teacher quality, and academic engagement, and four of the six factors were negatively associated with anxiety and ADHD symptoms. This supports previous work finding SEA as a protective factor in psychological and academic wellbeing (Kim et al., 2024).

As SEA has been associated with academic engagement in elementary and secondary school students (Durlak et al., 2011), an aim of this study was to investigate the role of SEA as a unique predictor of academic engagement in university students. After accounting for perseverance, anxiety, and student perceptions of teacher quality, empathy was found to be a unique predictor of behavioral, emotional, and cognitive engagement. This supports previous work which has found perspective taking, theory of mind and empathy predict academic engagement (Bosacki et al., 2019). In addition, adaptability was found to be a unique predictor of emotional and cognitive engagement, which is consistent with work on growth mindset, academic commitment, and effortful engagement (Tang et al., 2019). Finally, assertiveness was a unique predictor of behavioral and cognitive engagement which replicates work students' confidence and agency as a predictor of engagement (Reeve, 2013).

### Limitations

This work was conducted in western Canada, at a public university over the duration of 4 years. Cohort effects were found, with students in the first wave of data reporting lower levels of SEA as compared to subsequent waves. Data were collected when university courses were in person, prior to and after the COVID-19 pandemic (winter 2018, winter 2019, fall 2019, fall 2022). However, global events may have impacted students' responses.

Although efforts were made to include focus group and survey participants from diverse backgrounds, immigration, and economic status, all participants were English speaking in a Canadian context. Therefore, usage of the SEA measure in other contexts is required to investigate the structural validity and associations with psychological and academic wellbeing. Although 66% of participants were ethnic minorities, comparisons could not be made across these ethnic groups due to demographic imbalance.

A large majority of survey participants were women. This reflects the local university population in which the study was conducted, particularly among introductory psychology students. However, future work is needed to focus on the unique role of masculinity on social emotional development in emerging adulthood. As more young men are reporting feelings of isolation, burnout, and frustration (Sparks et al., 2024), identification of protective factors could help to ameliorate risks to young men's wellbeing.

In addition, sampling from introductory psychology classes may have oversampled particularly socially and emotionally aware individuals. Future work is needed to recruit young adults from other university majors, and from outside of higher education to compare differences in social emotional development in emerging adulthood in different career and education trajectories. Therefore, the generalizability of this work is limited to primarily women in higher education with an interest in psychology, and current findings may not be applicable to other demographics.

The instrument developed in this work has several drawbacks. The SEA measure relies on self-report and is therefore vulnerable to social desirability bias. Therefore, individuals may be rating themselves as higher in these competencies than they would otherwise admit or may have a conflated view of their social and emotional competence. Future work is needed to cross-validate the SEA measure with observational, dyadic reporting, or other metrics of social and emotional competency.

Despite efforts to remove redundant questionnaire items at the outset of the measurement design, several similar items were listed on the version of the SEA measure distributed to participants. This impacted the exploratory factor analysis, in that similar items on considering outcomes and gratification delay loaded as separate Factors 6 and 7. Although these were theoretically combined as the factor of Prudence, internal reliability for this factor was poor, and any significant findings regarding this factor should be interpreted with caution.

To further distinguish SEA from indices of personality, intervention or longitudinal work is needed. Although short term interventions on university students in Canada have found improvement in SEA (Vargas-Madriz and Konishi, 2025), the ability to track changes and maturity in both SEA and personality overtime is needed. As social competence continues to develop in middle and late adulthood (Rakoczy et al., 2018), cross-section work is needed to examine how SEA might vary as a function of age in young, middle, and older adulthood.

### Implications

The identification of the six-factor structure (empathy, cooperation, adaptability, awareness, assertiveness, prudence) and the development of the SEA scale may benefit from future research using this measure in diverse college student and early adulthood populations. In particular, cross-cultural replication of this measure is needed, along with further validation from longitudinal rather than cross-sectional work.

SEA has been connected to increased psychological wellbeing, academic wellbeing, and educational attainment (Elias and Weissberg, 2000). Moreover, long-term outcomes of SEA have included reduced job burnout, success in finding employment, and increased relationship satisfaction (Denham, 2018). In terms of life skills that promote health, happiness, and success, social and emotional competence may be equally as vital as numeracy and literacy (Kim et al., 2024). Therefore, future work is needed to determine if fostering these competencies in higher education may assist with student mental health, academic achievement, and preparation for both healthy relationships and the labor market. Although many individuals develop SEA implicitly and through social exposure, others may benefit from explicit instruction and intervention strategies that help to comprehend and model social and emotional skills.

## Conclusion

Overall, this study aimed to develop a measure of Social Emotional Assets (SEA) for emerging adults that reduced cultural bias. This measure was found to have appropriate convergent and divergent validity with personality, anxiety, ADHD, and perseverance. In addition, subcomponents of this measure were found to be unique and significant predictors of student engagement in university.

## Data Availability

The raw data supporting the conclusions of this article will be made available by the authors, without undue reservation.

## Appendix A

**Table:** Questionnaire items removed during focus group.

Removed due not focusing on interpersonal or self-regulatory skills I know which skills I perform well and which skills I do not I know how to revise and improve my work I am able to juggle multiple demands I adapt my overall strategies, goals, or projects to fit the situation I handle my belongings with care I seek to improve myself by setting measurable and challenging goals
Removed due to lack of cultural universality in social norms I look at people when I talk to them I enjoy greeting and introducing myself to others I carry myself with confidence I keep my promises I am comfortable trusting my friends with private information I try to help everyone participate positively in an activity I often help to facilitate cooperation amongst others I am skilled at making others feel better when they are upset I tell people when I have made a mistake I accept responsibility for my actions I resolve conflict by bringing it into the open I use my past mistakes to make better choices I understand the reasons for others' actions When other people pressure me, I don't let it bother me I understand the opinions of others I do not understand the subtle feelings of others I understand how people expect me to behave I can block out distractions when working
Removed due to age inappropriateness in emerging adulthood I rarely get into trouble for being careless I do the right thing without be told I follow directions in class I feel bad when others are sad I attract positive attention from my peers I cooperate with peers and siblings I can remember important information I try to do things independently I say good things about my classmates

## References

[B1] Arnett J. J. (2024). Emerging Adulthood: The Winding Road from the Late Teends Through the Twenties, 3rd Edn. New York, NY: Oxford University Press. doi: 10.1093/oso/9780197695937.001.0001

[B2] Ashton M. C. Lee K. (2007). Empirical, theoretical, and practical advantages of the HEXACO model of personality structure. Pers. Soc. Psychol. Rev. 11, 150–166. doi: 10.1177/1088868306294907 18453460

[B3] Ashton M. C. Lee K. (2020). Objections to the HEXACO model of personality structure – and why those objections fail. Eur. J. Pers. 34, 492–510. doi: 10.1002/per.2242

[B4] Barth K. M. Tepelea M. Racasan R. (2025). Linking self-reported symptoms of ADHD, dyslexia, and emotional distress to academic engagement in students. Educatia 21, 63–74. doi: 10.24193/ed21.2025.31.07

[B5] Bierman K. L. Motamedi M. (2015). “SEL programs for preschool children,” in Handbook of Social Emotional Learning: Research and Practice, eds J. A. Durlak, C. E. Domitrovich, R. P. Weissberg, and T. P. Gullotta (New York, NY: Guilford Press), 135–150.

[B6] Boateng G. O. Neilands T. B. Frongillo E. A. Melgar-Quinonez H. R. Young S. L. (2018). Best practices for developing and validating scales for health, social, and behavioral research: a primer. Front. Public Health 6:149. doi: 10.3389/fpubh.2018.00149 29942800 PMC6004510

[B7] Bosacki S. Moreira F. Sitnik V. Andrews K. Talwar V. (2019). Theory of mind, emotion knowledge, and school engagement in emerging adolescents. J. Elem. Educ. 11, 529–538. doi: 10.26822/iejee.2019553349

[B8] Boyatzis R. E. Goleman D. (2007). Emotional and Social Competency Inventory. Boston, MA: Hay Group.

[B9] Chen Y. Wang H. Jiang Y. Zhang H. Chen C. (2025). Social emotional learning competencies and their interaction with perceived attachments to parents and peers on Chinese adolescent academic burnout. Sch. Psychol. Rev. 54. doi: 10.1080/2372966X.2025.2548760

[B10] Chopik W. J. Nuttall A. K. Oh J. (2022). Relationship-specific satisfaction and adjustment in emerging adulthood: the moderating role of adult attachment orientation. J. Adult Dev. 29, 40–52. doi: 10.1007/s10804-021-09380-6 35342275 PMC8942393

[B11] Christov-Moore L. Simpson E. A. Coude G. Grigaityte K. Iacoboni M. Ferrari P. F. (2014). Empathy: gender effects in brain and behaviour. Neurosci. Biobehav. Rev. 46, 604–627. doi: 10.1016/j.neubiorev.2014.09.001 25236781 PMC5110041

[B12] Coelho V. Peixoto C. Azevedo H. Machado F. Soares M. Espain A. (2023). Effects of a Portuguese social emotional learning program on the competencies of elementary school students. Front. Psychol. 14*:*1195746. doi: 10.3389/fpsyg.2023.1195746 37265946 PMC10230249

[B13] Conley C. S. (2015). “SEL in higher education,”. in Handbook of Social and Emotional Learning: Research and Practice, eds J. A. Durlak, C. E., Domitrovich, R. P. Weissberg, and T. P. Gullotta (New York, NY: Guilford Press), 197–212.

[B14] Costa P. T. McCrae R. R. (1995). Domains and facets: hierarchical personality assessment using the revised NEO personality inventory. J. Pers. Assess. 64, 21–50. doi: 10.1207/s15327752jpa6401_2 16367732

[B15] Denham S. A. (2018). Keeping SEL Development: The Importance of a Developmental Lens for Fostering and Assessing SEL Competences. Measuring SEL: Using Data to Inspire Practice. CASEL, Chicago.

[B16] Dhira T. A. Rahman M. A. Razzaque Sarker A. Mehareen J. (2021). Validity and reliability of the generalized anxiety disorder-7 (GAD-7) among university students of Bangladesh. PLoS ONE 16:34914811. doi: 10.1371/journal.pone.0261590 34914811 PMC8675645

[B17] Duckworth A. L. Peterson C. Matthews M. D. Kelly D. R. (2007). Grit: perseverance and passion for long-term goals. J. Pers. Soc. Psychol. 92, 1087–1101. doi: 10.1037/0022-3514.92.6.1087 17547490

[B18] Dunn T. J. Baguley T. Brunsden V. (2014). From alpha to omega: a practical solution to the pervasive problem of internal consistency estimation. Br. J. Psychol. 105, 399–412. doi: 10.1111/bjop.12046 24844115

[B19] Durlak J. A. Weissberg R. P. Dymnicki A. B. Taylor R. D Schellinger K. B. (2011). The impact of enhancing students' social and emotional learning: a meta-analysis of school-based universal interventions. Child Dev. 82, 405–432. doi: 10.1111/j.1467-8624.2010.01564.x 21291449

[B20] Elias M. Zins J. Weissburg R. Frey K. Greenberg M. Haynes N. . (1997). Promoting Social and Emotional Learning: Guidelines for Educators. Alexandria, VA: Association for Supervision and Curriculum Development.

[B21] Elias M. J. Weissberg R. P. (2000). Primary prevention: educational approaches to enhance social and emotional learning. J. School Health 70, 186–190. doi: 10.1111/j.1746-1561.2000.tb06470.x 10900595

[B22] Elmi C. (2020). Integrating social emotional learning strategies in higher education. Investig. Health Psychol. Educ. 10, 848–858. doi: 10.3390/ejihpe10030061 34542515 PMC8314289

[B23] Gardner H. (2011). Frames of Mind: The Theory of Multiple Intelligences (10th Anniversary ed.). New York, NY: Basic Books.

[B24] Goleman D. (1995). Emotional Intelligence. New York, NY: Bantam.

[B25] Gresham F. M. Elliott S. N. (2017). Social Skills Improvement System—Social-Emotional Learning Edition Rating Forms. Bloomington, MN: Pearson Assessments. doi: 10.1037/t80391-000

[B26] Hughes K. Coplan R. J. (2018). Why classroom climate matters for anxious solitary children: a study of differential susceptibility. *Sch. Psychol. Q*. 33, 94–102. 28318284 10.1037/spq0000201

[B27] Jackson D. Prochnow T. Ettekal A. V. (2024). Programs promoting physical activity and social- emotional learning for adolescents: a systematic literature review. J. School Health 94, 994–1004. doi: 10.1111/josh.13486 38962813

[B28] Jaramillo-Rincon S. X. Vazquez-Pena F. Rodriguez L. Trujillo-Maza E. (2024). Cross-cultural and psychometric validation of the “GRIT-S” scale for measuring academic tenacity in medical students in Spanish. Cogent. Educ. 12:2357921. doi: 10.1080/2331186X.2024.2357921

[B29] Katsiaficas D. (2017). I know I'm an adult when … I can care for myself and others: the role of social responsibilities in emerging adulthood for community college students. Emerg. Adulthood 5, 392–405. doi: 10.1177/2167696817698301

[B30] Kessler R. C. Alder L. A. Ames M. Demler O. Faraone S. Hiripi E. . (2005). The World Health Organization Adult ADHD Self-Report Scale (ASRS): a short screening scale for use in the general population. *Psychol. Med*. 35, 245–256. 15841682 10.1017/s0033291704002892

[B31] Kim E. K. Allen J. P. Jimerson S. R. (2024). Supporting student social emotional learning and development. School Psych. Rev. 53, 201–207. doi: 10.1080/2372966X.2024.2346443

[B32] Kline R. B. (2023). Principles and Practice of Structural Equation Modeling, 5th Edn. New York, NY: The Guilford Press.

[B33] Kohlberg L. (1984). The Psychology of Moral Development: The Nature and Validity of Moral Stages. San Franciso, CA: Harper and Row.

[B34] Krull D. S. Loy M. H. Lin J. Wang C. Chen S. Zhao X. (1999). The fundamental attribution error: correspondence bias in individualist and collectivist cultures. Pers. Soc. Psychol. Bull. 25, 1208–1219. doi: 10.1177/0146167299258003

[B35] Lahdelma P. Tolonen M. Kiuru N. Hirvonen R. (2021). The role of adolescents' and their parents' temperament types in adolescents' academic emotions: a goodness-of-fit approach. Child Youth Care Forum 50, 471–492. doi: 10.1007/s10566-020-09582-1

[B36] Lam S. Jimerson S. Wong B. Kikas E. Shin H. Veiga F. H. . (2014). Understanding and measuring student engagement in school: the results of an international study from 12 countries. School Psychol. Q. 29, 213–132. doi: 10.1037/spq0000057 24933218

[B37] Law Y. (2011). The role of teachers' cognitive support in motivating young Hong Kong Chinese children to read and enhancing reading comprehension. Teach. Teach. Educ. 27, 73–84. doi: 10.1016/j.tate.2010.07.004

[B38] Lawson L. (2023). Factor structure and instrument stability of the DESSA-Mini: an evaluation of the gender, race, and grade category of an SEL Screening Assessment. J. Psychoeduc. Assess. 41, 1001–1015.

[B39] Leaper C. Ayres M. M. (2007). A meta-analytic review of gender variations in adults' language use: talkativeness, affiliative speech, and assertive speech. Pers. Soc. Psychol. Rev. 11, 328–363. doi: 10.1177/1088868307302221 18453467

[B40] LeBuffe P. A. Shapiro V. B. Naglieri J. A. (2014). The Devereux Student Strengths Assessment (DESSA): Assessment, Technical Manual, and User's Guide. Charlotte, NC: Apperson, Inc. ProQuest Dissertations and Theses. doi: 10.1037/t15187-000

[B41] Lee K. Ashton M. C. (2004). Psychometric properties of the HEXACO personality inventory. Multivar. Behav. Res. 39, 329–358. doi: 10.1207/s15327906mbr3902_8 26804579

[B42] Lee N. Y. W. Zhang M. W. B. (2025). Systematic review on prevalence of ADHD, possible ADHD, or ADHD symptoms in medical students. Front. Psychiatry 16:1684727. doi: 10.3389/fpsyt.2025.1684727 41409340 PMC12706586

[B43] Lee S. A. (2009). Does empathy mediate the relationship between neuroticism and depressive symptomatology among college students? Pers. Individ. Dif. 47, 429–433. doi: 10.1016/j.paid.2009.04.020

[B44] Li A. Miller F. G. Williams S. C. (2025). Cultural adaptations to social-emotional learning programs: a systematic review. School Psychol. 40, 108–120. doi: 10.1037/spq0000649 39052395

[B45] Li L. Li Y. Zheng X. (2025). Assertiveness or humility? The role of broker's humility in promoting knowledge-based gig employee's organizational identity and proactive behaviour. Balt. J. Manag. 20, 282–302. doi: 10.1108/BJM-10-2024-0695

[B46] Malcolm C. Saxton T. K. McCarty K. Roberts S. G. B. Pollet T. V. (2021). Extraversion is associated with advice network size, but not network density or emotional closeness to network members. Pers. Individ. Dif. 168:110311. doi: 10.1016/j.paid.2020.110311

[B47] Manian S. Sheth K. (2021). Follow my lead: assertive cheap talk and the gender gap. Manage. Sci. 67, 6880–6896. doi: 10.1287/mnsc.2020.3837

[B48] McAbee S. T. Casillas A. Way J. D. Guo F. (2019). The HEXACO model in education and work. current applications and future directions. Z. Psychol. 227, 174–185. doi: 10.1027/2151-2604/a000376

[B49] McCall C. S. Romero M. E. Yang W. Weigand T. (2023). A call for equity-focused social-emotional learning. School Psych. Rev. 52, 586–607. doi: 10.1080/2372966X.2022.2093125

[B50] McCarthy A. Lee K. Itakura S. Muir D. W. (2006). Cultural display rules drive eye gaze during thinking. J. Cross Cult. Psychol. 37, 717–722. doi: 10.1177/0022022106292079 19122788 PMC2613330

[B51] Merrell K. W. Juskelis M. P. Tran O. K. Buchanan R. (2008). Social and emotional learning in the classroom: evaluation of strong kids and strong teens on students' social-emotional knowledge and symptoms. J. Appl. Sch. Psychol. 1, 209–224. doi: 10.1080/15377900802089981

[B52] Mills S. D. Fox R. S. Malcarne V. Rosech S. C, Champagne, B. R. Sadler G. R. (2014). The psychometric properties of the general anxiety disorder 7-scale. Cultur. Divers. Ethnic. Minor. Psychol. 20, 463–468. doi: 10.1037/a0036523 25045957 PMC4129392

[B53] Morgado F. F. R. Meireles J. F. F. Neves C. M. Amarai A. C. S. Ferreira M. E. C. (2017). Scale development: ten main limitations and recommendations to improve future research practices. Psicol.: Reflex. Crit. 30:3. doi: 10.1186/s41155-016-0057-1 32025957 PMC6966966

[B54] Ng P. Y. Yang S. Chiu R. (2024). Features of emerging adulthood, perceived stress and life satisfaction in Hong Kong emerging adults. Curr. Psychol. 43, 20394–20406. doi: 10.1007/s12144-024-05811-1

[B55] Ntum S. Kwaku K. C. Agyemang K. Nyarko B. (2025). Assessing what matters: integrating social-emotional learning into classroom assessment for pupil well-being in Ghanaian basic schools. Nat. Sci. Rep. 15:45127. doi: 10.1038/s41598-025-33328-5 41429900 PMC12749574

[B56] OECD (2018). Social and Emotional Skills for Student Success and Well-being: Conceptual Framework for the OECD Study on Social and Emotional Skills. Paris: OECD Education Working Papers No. 173.

[B57] Panorama Education (2020). Reliability and Validity of Panorama's Survey Topics for Students: 2020 Update. Boston, MA: Panorama Education.

[B58] Poonamallee L. Harrington A. M. Nagpal M. Musial A. (2018). Improving emotional intelligence through personality development: the effect of smart phone application based Dharma life program on emotional intelligence. Front. Psychol. 9:169. doi: 10.3389/fpsyg.2018.00169 29527182 PMC5829461

[B59] Putnick D. L. Bornstein M. H. (2016). Measurement invariance conventions and reporting: the state of the art and future directions for psychological research. Develop. Rev. 41, 71–90. doi: 10.1016/j.dr.2016.06.004 27942093 PMC5145197

[B60] Rakoczy H. Wandt R. Thomas S. Nowak J. Kunzmann U. (2018). Theory of mind and wisdom: the development of different forms of perspective-taking in late adulthood. Br. J. Psychol. 109, 6–24. doi: 10.1111/bjop.12246 28266717

[B61] Reeve J. (2013). How students create a motivationally supportive learning environments for themselves: the concept of agentic engagement. J. Educ. Psychol. 105, 579–595. doi: 10.1037/a0032690

[B62] Schafer J. L. Graham J. W. (2002). Missing data: our view of the state of the art. Psychol. Methods 7, 147–177. doi: 10.1037/1082-989X.7.2.147 12090408

[B63] Shi J. Cheung A. C. K. (2024). Effective components of social emotional learning programs: a meta-analysis. J. Youth Adolesc. 53, 755–771. doi: 10.1007/s10964-024-01942-7 38280178

[B64] Sigmundsson H. (2021). Passion, grit, and mindset in the ages 14 to 77: exploring relationships and gender differences. New Ideas Psychol. 60:100815. doi: 10.1016/j.newideapsych.2020.100815

[B65] Solmi M. Radua J. Olivola M. Croce E. Soardo L. de Pablo G. S. . (2022). Age at onset of mental disorders worldwide: large-scale meta-analysis of 192 epidemiological studies. Mol. Psychiatry 27, 281–295. doi: 10.1038/s41380-021-01161-7 34079068 PMC8960395

[B66] Sparks B. Zidenberg A. M. Olver M. E. (2024). One is the loneliest number: involuntary celibacy. Curr. Psychol. 43:392. doi: 10.1007/s12144-023-04275-z 36747916 PMC9892684

[B67] Stefan C. A. Danila I. Cristescu D. (2022). Classroom-wide school interventions for preschoolers' social-emotional learning: a systematic review of evidence-based programs. Educ. Psychol. Rev. 34, 2971–3010. doi: 10.1007/s10648-022-09680-7

[B68] Tabassum R. Akhter N. Iqbal Z. (2020). Relationship between social competence and academic performance of university students. J. Educ. Res. 23:111.

[B69] Tang X. Wang M. Guo J. Salmela-Aro K. (2019). Building grit: the longitudinal pathways between mindset, commitment, grit, and academic outcomes. J. Youth Adolesc. 48, 850–863. doi: 10.1007/s10964-019-00998-0 30788767

[B70] Tosi G. Romano D. Besson T. De Houwer J. Nevejans M. Perugini M. (2025). The HEXACO adjective scale: a cross-cultural validity study. J. Pers. Assess. 11, 1–10. doi: 10.31219/osf.io/pvk6u_v1 41268970

[B71] Vargas-Madriz L. F. Konishi C. (2025). Promoting social inclusion, school engagement and reducing stress among newcomer university students: an online social-emotional learning pilot programme. J. Appl. Res. High. Educ. 18. doi: 10.1108/JARHE-11-2024-0596

[B72] Worthington R. L. Whittaker T. A. (2006). Scale development research: a content analysis and recommendations for best practices. *Couns. Psychol*. 34, 806–838.

[B73] Yang C. Golshirazi M. (2024). Association between school victimization and substance use among Hispanic/Latinx adolescents: an intersectionality analysis of social-emotional learning (SEL) competencies, immigration status, and gender in predominantly Hispanic/Latinx high schools. School Psych. Rev. 53, 552–564. doi: 10.1080/2372966X.2022.2158368

[B74] Yang X. Guan Y. Zhang Y. She Z. Buchtel E. E. Mak M. C. K. . (2020). A relational model of career adaptability and career prospects: the roles of leader-member exchange and agreeableness. J. Occup. Organ. Psychol. 93, 405–430. doi: 10.1111/joop.12301

[B75] Yeh E. Sharma R. Jaiswal-Oliver M. Wan G. (2022). Culturally responsive social emotional learning for international students: professional development for higher education. J. Int. Stud. 12, 19–41. doi: 10.32674/jis.v12i1.2976

[B76] Zhang J. Liu C. (2025). Bidirectional and longitudinal associations among teacher-student relationships, peer relationships, and learning engagement in Chinese primary school students: a cross-lagged panel model. Front. Psychol. 16:1674600. doi: 10.3389/fpsyg.2025.1674600 41356057 PMC12679389

